# Bayesian methods in clinical trials: a Bayesian analysis of ECOG trials E1684 and E1690

**DOI:** 10.1186/1471-2288-12-183

**Published:** 2012-11-29

**Authors:** Joseph G Ibrahim, Ming-Hui Chen, Haitao Chu

**Affiliations:** 1 Department of Biostatistics, University of North Carolina, McGavran Greenberg Hall, CB#7420, Chapel Hill, NC, 27599, USA; 2 Department of Statistics, University of Connecticut, Storrs, CT, USA; 3 Division of Biostatistics, University of Minnesota, Minneapolis, MN, USA

**Keywords:** Cure rate model, Historical data, Prior distribution, Posterior distribution

## Abstract

**Background:**

E1684 was the pivotal adjuvant melanoma trial for establishment of high-dose interferon (IFN) as effective therapy of high-risk melanoma patients. E1690 was an intriguing effort to corroborate E1684, and the differences between the outcomes of these trials have embroiled the field in controversy over the past several years. The analyses of E1684 and E1690 were carried out separately when the results were published, and there were no further analyses trying to perform a single analysis of the combined trials.

**Method:**

In this paper, we consider such a joint analysis by carrying out a Bayesian analysis of these two trials, thus providing us with a consistent and coherent methodology for combining the results from these two trials.

**Results:**

The Bayesian analysis using power priors provided a more coherent flexible and potentially more accurate analysis than a separate analysis of these data or a frequentist analysis of these data. The methodology provides a consistent framework for carrying out a single unified analysis by combining data from two or more studies.

**Conclusions:**

Such Bayesian analyses can be crucial in situations where the results from two theoretically identical trials yield somewhat conflicting or inconsistent results.

## Background

### Brief Introduction to Bayesian Methods

Bayesian methods in clinical trials and biomedical research, in general, have become quite prominent in the last decade due to their flexibility in use, good operating characteristics, interpretation, and in their ability to handle design and analysis issues in complex models, such as survival models, models for longitudinal data, and models for discrete data. Bayesian methods are becoming more and more standard in the design and analysis of clinical trials [1,2]. One main reason for this is in their flexibility and operating characteristics, for example, in adaptive designs and interim monitoring [2]. There are many good introductory as well as advanced level books on Bayesian methods [3-5].

The Bayesian paradigm differs from the frequentist paradigm in that our uncertainty about unknown parameters in a model is expressed through an entire distribution, called the *prior distribution*. Thus, the prior distribution for a model parameter θ, expresses our prior uncertainty about the value of the parameter, and “prior” in the sense that we express our uncertainty before collecting the data in the study. We denote the prior distribution for θ by *π*(*θ*). The main inferential tool in the Bayesian paradigm is called the posterior distribution, that is, the distribution of *θ* after the data is collected. By Bayes’ theorem, the posterior distribution of θ is proportional to the likelihood function of the data times the prior. In terms of a formula, it is given by *π*(*θ*∣Data) ∝ *L*(Data∣*θ*)*π*(*θ*), where *π*(*θ*∣Data) denotes the posterior distribution of *θ* that is, the distribution of θ *given* the data, and *L*(Data∣*θ*) denotes the likelihood function of the data given the parameter *θ*.

A major consideration in the Bayesian paradigm is the choice of the prior. Priors that have a minimal impact on the overall Bayesian analysis are called *noninformative* priors. Other names for noninformative priors include flat, reference, or vague priors. Noninformative priors yield Bayesian inferences that are very similar to frequentist inference. A noninformative prior is “flat” relative to the likelihood function, that is, it is flat relative to the distribution of the data. On the other hand, informative priors generally do not lead to results that are similar to those of the frequentist paradigm and such priors are not flat relative to the likelihood function and do have an impact on the likelihood in a Bayesian analysis. Examples of potentially informative priors used in biomedical research include priors incorporating historical data [6,7].

Bayesian models can be fit in a wide variety of statistical packages including WinBUGS and SAS. These software packages have become very powerful in providing the data analyst with a wide array of flexibility and capability for fitting complex Bayesian models. Both of these software packages use Markov chain Monte Carlo (MCMC) methods to carry out the Bayesian computation. MCMC methods are simulation-based methods that draw samples from the posterior distribution of *θ* and have proven to be quite powerful for fitting even the most complex models that cannot be entertained in the frequentist paradigm.

### Brief Introduction to Melanoma

Melanoma incidence is increasing at a rate that exceeds all solid tumors. Although education efforts have resulted in earlier detection of melanoma, patients who have deep primary melanoma (*>*4*mm*) or melanoma metastatic to regional draining lymph nodes, classified as *high-risk melanoma* patients, continue to have high relapse and mortality rates of 50% or higher [8]. Recently, several post-operative (adjuvant) chemotherapies have been proposed for this class of melanoma patients, and the one which seems to provide the most significant impact on relapse-free survival and survival is Interferon Alpha-2b (IFN). This immunotherapy was evaluated in two observation-controlled Eastern Cooperative Oncology Group (ECOG) phase III clinical trials, E1684 and E1690. The first trial, E1684, was a two arm clinical trial comparing high-dose interferon (IFN) to Observation (OBS). There were a total of 286 patients enrolled in the study, accrued from 1984 to 1990, and the study was unblinded in 1993 and published in 1996 [8]. The results of this study suggested that IFN has a significant impact on relapse-free survival and survival, which led to the U.S. Food and Drug Administration (FDA) approval of this regimen as an adjuvant therapy for high-risk melanoma patients. Here, relapse-free survival is defined as the time from randomization until progression of tumor or death, whichever comes first, and survival is defined as time from randomization until death. This regimen is widely used for adjuvant therapy of high-risk melanoma patients and the reference standard for evaluation of alternative modalities such as vaccines in current U.S. Cooperative group trials.

The significant treatment effect favoring IFN seen in E1684 with respect to both relapse- free survival (RFS) and overall survival (OS) was expected and was accompanied by substantial side effects due to the high-dose regimen. As a result, ECOG began a second trial (E1690) in 1991 to attempt to confirm the results of E1684 and to study the potential benefit of IFN given at a lower and less toxic dosage. The ECOG trial E1690 was a three arm phase III clinical trial, and had treatment arms of high dose interferon, low dose interferon, and observation. This study had 427 patients on the high dose interferon arm and observation arm combined. Throughout our analyses in this paper, we will use only the data from these two arms of E1690. E1690 was initiated right after the completion of E1684. The E1690 trial accrued patients from 1991 until 1995, was unblinded in 1998, and published in 2000 [9]. The E1690 trial was designed for exactly the same patient population as E1684, and the high dose interferon arm in E1690 was identical to that of E1684.

E1690 was a critical trial in the assessment of the value of high-dose IFN as adjuvant therapy for melanoma. When the results of E1690 were unblinded, separate results for E1684 and E1690 were reported [9], and analyses of the combined results were problematic and unable to be resolved into one coherent analysis. In this paper, we propose to do a combined analysis of the E1684 and E1690 trials using Bayesian methods. The Bayesian methodology lends itself well into this type of analysis since the E1684 data can effectively be used as prior information for the E1690 analysis. Using the E1684 data as the historical data and the E1690 data as the current data is natural here within the Bayesian paradigm and this will serve as the basis for our analysis. We thus examine the problem of developing suitable statistical models for high-risk melanoma patients as well as the opportunity of conducting Bayesian inference in the presence of historical data. In this article, we will discuss a Bayesian analysis for the endpoints of RFS as well as OS. It was in the OS endpoint where the results were most inconsistent between the two trials and it was this endpoint which has led to most of the controversy. The RFS results were more or less consistent between E1684 and E1690. The results of this trial also raised the issue of whether RFS can be used at all as a suitable surrogate or predictor of OS, since the RFS and OS results were not consistent in E1690.

In the present context, the incorporation of historical data, i.e., the E1684 data, into the analysis of E1690 is a natural thing to do. Towards this goal, we use the power priors of Ibrahim and Chen [6] to construct the prior distribution. Since the FDA has in the past often required a second confirmatory trial before approving a new drug for cancer therapy, historical data often exists for constructing prior distributions in a clinical trial from a previous trial or trials comparing identical or very similar treatment regimens. Such is the case for the E1684 and E1690 trials. Thus, it appears natural to use the E1684 data somehow for the analysis of E1690.

The construction of prior distributions from historical data has been examined under various contexts by Ibrahim and Chen [6]. Where the focus is on observable quantities in the prior elicitation scheme. Specifically, prior elicitation is based on the availability of historical data *D*_0_ and a scalar *precision* parameter *a*_0_ (0 *≤ a*_0_ *≤* 1) quantifying the uncertainty in *D*_0_. Then, *D*_0_ and *a*_0_ are used to specify a prior distribution for the parameters in a “semi-automatic” fashion. Strictly speaking, *D*_0_ can consist of prior predictions from past data, summary statistics from previous studies, or subjective elicitation based on case-specific information. However, the most natural specification of *D*_0_ arises when the raw data from a similar previous study is available, and this is what we focus on in this paper. There are many advantages to this type of elicitation scheme. First, the prior is constructed in a semi-automatic fashion from the historical data in the sense that the prior itself is just a weighted likelihood and thus there is minimal subjective prior elicitation. Second, the precision parameter *a*_0_ allows the investigator a great deal of control on the influence of the historical data for the current analysis. This is important in situations when one suspects heterogeneity between the patient populations, or when the sample sizes between the two studies are quite different.

Thus, in the analysis we present here, there are several key issues we address. These are

(a) Patient heterogeneity between the two studies.

(b) Methods for incorporation of historical data.

(c) Consistency of the results from the two studies.

(d) A pooled analysis.

(e) Assessing Observation and high-dose IFN time trends.

As a result of these issues, several questions arise that we also address here. These are

(1) Should the analysis of E1690 be done independently of E1684?

(2) If we are interested in the treatment of melanoma, how can E1864 be used as historical data for E1690?

(3) How does E1684 impact the results?

(4) Can we assess and control the impact of E1684 by weighting it somehow?

(5) How do we weight the historical data to account for heterogeneity between the two studies?

## Methods

### Cure Rate Model

**Figure 1:**
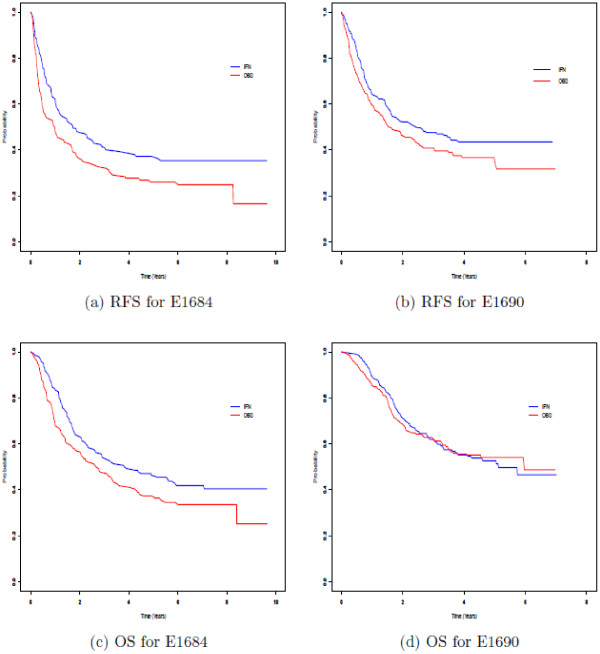
Kaplan-Meier RFS plots for E1684 (a) and E1690 (b) and Kaplan-Meier OS plots for E1684 (c) and E1690 (d).

We focus on a Bayesian analysis using a cure rate model for these data. Details of the cure rate model are described in [5]. The cure rate model has been a key component in the design of adjuvant melanoma ECOG trials, and this model was used to design E1690, E1694, and the E1697 adjuvant melanoma trials. The cure rate model is a useful model for designing studies with time-to-event endpoints, such as RFS and OS. It is most useful when a plateau is reached in the survival curve after sufficient follow-up. For adjuvant melanoma studies, this plateau occurs after about 5 years based on the ECOG experience. To illustrate this plateau, we consider an RFS plot for E1684 at 6.96 years of follow-up. From the plot shown in Figure 1a, we see that the plateau begins to occur after 5 years of follow-up.

In the cure rate model, we assume that our population really consists of two subpopulations: cured and non-cured. We let *π* = proportion of patients in the population who are cured, and 1 *− π* = proportion of patients in the population who are not cured. The proportion not cured experience events according to a survival function S_0_(t) = P(T > t). Then the probability of surviving beyond time *t* for the entire population (i.e., the survival function for the entire population), denoted by S_p_(t), is given by

(1) $$S_{p}\left(t\right)=\pi +\left(1-\pi \right)S_{0}\left(t\right)$$

For example, *π* = 0*.*25 means that 25% of the population are “cured” and 75% are “not cured”. If *π* = 0, then we obtain the survival model with survival function S_0_(t). The cure rate model fits the data better than the usual Cox model when a plateau occurs in the right tail of the survival curve, and thus, for E1684, and most other melanoma adjuvant trials in ECOG, the cure rate model fits the data better than the Cox model. The cure rate model also has other attractive properties. For example, the log-rank test has nice properties (i.e., high power) when a cure rate model is used in the statistical design [9,10]. Also, the cure rate model is quite easy to fit and computationally very straightforward to program in SAS or R.

### Prior Elicitation

The specification of the prior distribution is called *prior elicitation*. Prior elicitation plays the most crucial role in Bayesian inference. Prior distributions based on historical data are useful in applied research settings where the investigator has access to previous studies measuring the same response and covariates as the current study.

Plots (a) and (b) in Figure 1 show the Kaplan-Meier survival curves by treatment arm for E1684 and E1690 for the RFS endpoint and Plots (c) and (d) in Figure 1 show the curves for the OS endpoint based on the analysis and follow-up reported in Kirkwood et al. [9]. An interesting phenomenon is observed. We see from these plots that, compared to E1684, there is an upward shift in both RFS and OS in E1690 for both of the treatment arms, and this shift is more pronounced in the observation arm. This feature in the Kaplan-Meier curves has been difficult to explain and was indeed an unexpected occurrence. This phenomenon raises the issue of whether the two studies in fact are comparable and have identical patient populations. A second striking feature occurs in Figure 1c and d, where the Kaplan-Meier curves for OS in E1690 are practically in complete overlap for the two treatment arms, while in Figure 1c, there is substantial separation between the two treatment arms in the E1684 study. Figure 1a and b on the other hand show that the RFS endpoint is essentially consistent for E1684 and E1690. Figure 1 has generated much discussion regarding the OS benefit of high-dose IFN, and whether the RFS endpoint is a worthwhile measure for melanoma. Many argue that RFS is of little value if it does not produce an OS benefit, while others argue that RFS is still useful in identifying treatment strategies for OS benefit. This controversy was hotly debated based on Figure 1. This controversy, however, is at least partially resolved in a combined analysis using the Bayesian methodology in Section 4. Figure 1 genuinely establishes the general importance of the issue of compatibility between the historical data and the current data. That is, if one is going to incorporate historical data into the current analysis, one must first investigate whether there is adequate homogeneity or “match” between the historical and current data in order to provide some justification for using the historical data in the current analysis. Although, as we will demonstrate, the lack of compatibility can be reflected by the choice of *a*_0_, it is desirable to have historical and current datasets with minimum heterogeneity between the patient populations. Although the E1684 and E1690 trials theoretically involved identical patient populations, Figure 1 suggests that one should formally check if the historical data, E1684, is compatible with the current data, E1690, in some sense. In the context of these two trials, there are several practical formal checks one can make given a statistical model. These are outlined as follows.

1. We compare important demographic and prognostic factor data from both studies, including distributions of gender, nodal status, age, Breslow depth, site of primary, ulceration, stage of disease, as well as other prognostic factors deemed important. Although not shown here, these comparisons were conducted for E1684 and E1690, and the distributions of these prognostic factors matched remarkably well. That is, the distributions of each of the demographic variables were nearly identical for both studies.

2. A more formal procedure involves a comparison of the posterior distributions for each study. We can compare posterior summaries, such as posterior hazard ratios, posterior standard deviations, and Highest Posterior Density (HPD) intervals.

**Figure 2:**
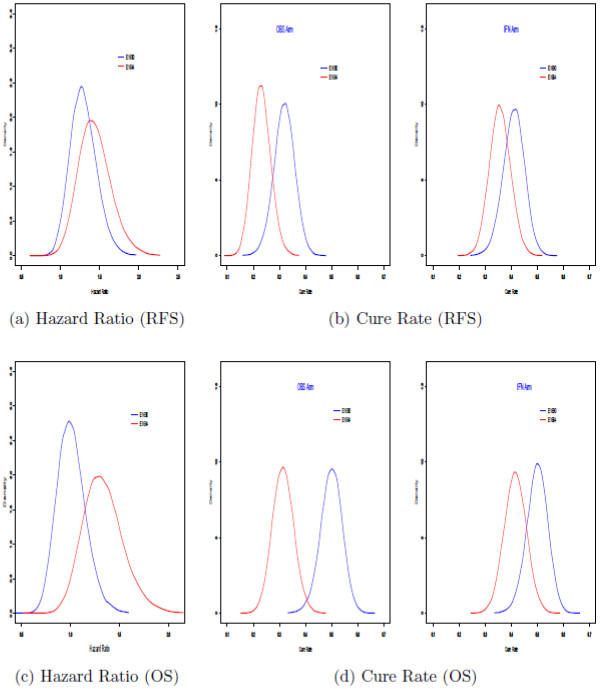
Plots of posterior densities of hazard ratios (a and c) and cure rates (b and d) for E1684 and E1690.

To examine the compatibility between both studies, several posterior summaries were computed. Table 1 summarizes the maximum likelihood estimates of the hazard ratios for both studies, where IFN was a reference arm in the hazard ratio calculation, as well as the maximum likelihood estimates of the cure rates. Figure 2 shows the posterior hazard ratios and the posterior distributions of the cure rates under model (A.1) in Additional file 1: Appendix A for E1684 and E1690 analyzed separately. We see from Table 1 and Figure 2 that the two studies are quite comparable, and there is a great deal of overlap between the posterior distributions of the hazard ratios for both studies. This overlap can also be seen from the 95% confidence intervals in Table 1. From these results, we can conclude that there is adequate compatibility between the E1684 data and the E1690 data, therefore justifying the use of the E1684 data as historical data to be incorporated into an informative prior distribution for a Bayesian analysis of E1690.

**Table 1:** Maximum likelihood estimates of hazard ratios and cure rates for E1684 and E1690

**Endpoint**	**Study**	**Hazard ratio**	**Cure rate**	
		**(95% CI)**	**(95% CI)**	
			**OBS**	**IFN**
RFS	E1684	1.43	0.17	0.35
		(1.08, 1.89)	(0.06, 0.33)	(0.28, 0.43)
	E1690	1.28	0.32	0.44
		(1.00, 1.65)	(0.24, 0.40)	(0.37, 0.50)
OS	E1684	1.32	0.25	0.40
		(0.98, 1.78)	(0.12, 0.41)	(0.32, 0.49)
	E1690	1.00	0.49	0.47
		(0.75, 1.33)	(0.36, 0.60)	(0.37, 0.56)

We use the *power prior* as discussed in [6] to formally construct an informative prior distribution from the historical data E1684. The general idea of the power prior is that it is defined to be the likelihood function based on the historical data *D*_0_, raised to a power *a*_0_, where 0 *≤ a*_0_ *≤* 1 is the scalar precision parameter that controls the influence of the historical data on the current data. Letting *θ* denote a generic label for the parameters of a given model, the general power prior for a model is defined as

(2) $$\pi \left(\theta |D_{0},a_{0}\right)\propto L\left(\theta |D_{0},a_{0}\right)\pi_{0}\left(\theta \right)$$

The quantity *π*_*0*_(*θ*) is the *initial prior* for *θ*. It represents the prior for *θ before* observing the historical data D_0_. It is reasonable to restrict the range of *a*_0_ to be between 0 and 1, since in general, it does not make sense to weight the historical data more than the current data. The parameter *a*_0_ controls the heaviness of the tails of the prior for *θ*. As *a*_0_ becomes smaller, the tails of equation (2) become heavier. Setting *a*_0_ = 1, equation (2) corresponds to the update of *π*_0_(*θ*) using Bayes theorem. That is, with *a*_0_ = 1, equation (2) corresponds to the posterior distribution of *θ* based on the historical data alone. When *a*_0_ = 0, equation (2) does not depend on the historical data, and in this case, *π*(*θ*|*D*_0_, *a*_0_ = 0) ≡ *π*_0_(*θ*). Thus, *a*_0_ = 0 is equivalent to a prior specification with no incorporation of historical data. Equation (2) can be viewed as a generalization of the usual Bayesian update of *π*_0_(*θ*), and therefore serves as a coherent update of *π*_0_(*θ*). Details of the power prior, likelihood, and posterior for the models used in this paper are given in Additional file 1: Appendix A. We mention here that there are two ways of specifying the power prior: i) using *a*_0_ as a fixed parameter and ii) treating *a*_0_ as random and specifying a beta prior for it as discussed in [6]. In our experience, we have observed that essentially similar results are obtained whether we take *a*_0_ as fixed or random. The *a*_0_ random case is more computationally intensive than the *a*_0_ fixed case and probably not worth the extra modeling and computation, and thus we advocate using *a*_0_ fixed and using a goodness of fit criterion (see Additional file 1: Appendix B) to select the best *a*_0_. Such a procedure is much less computationally demanding than the *a*_0_ random case.

## Results

### Combined Bayesian analysis of E1690 and E1684

In this section, we carry out a Bayesian analysis of E1690 using E1684 as historical data. For ease of exposition, all analyses are carried out with the treatment covariate alone, not adjusting for other prognostic factors. The two datasets were quite similar with respect to the distributions of several prognostic factors, including age, Breslow depth, number of nodes, performance status, gender, site of primary, and stage of disease. Prognostic factor analyses were conducted to examine the significance of time trend covariates and institutional effects for each study alone, as well as for the combined studies, for explaining the phenomenon in Figure 1. These factors were highly non-significant. We refer the reader to Kirkwood et al. [9] for detailed summaries of the prognostic factor distributions for both studies, as well as detailed prognostic factor analyses and various subset analyses conducted using the Cox model. The various prognostic factor and subset analyses in [9] yielded very similar results to analyses using the treatment covariate alone. In addition, Bayesian analyses with the proposed models using the prognostic factors mentioned above gave very similar results to the Bayesian analyses using the treatment covariate alone. Thus we conduct all analyses here with the treatment covariate alone for brevity.

We consider Bayesian analyses using the model in equation (A.1) in Additional file 1: Appendix A, in which the likelihood corresponds to a cure rate model along with a piecewise exponential model for the promotion time cumulative distribution function (cdf) (see [11]). The piecewise exponential model for the promotion time cdf is a very flexible model and it can accommodate any shape of the hazard function as well as being very computationally attractive. Our goal in this analysis is to combine the two studies using the power prior in equation (2), and provide a coherent and unified Bayesian analysis combining the data from both studies. As noted earlier, Table 1 shows estimates of hazard ratios and 95% confidence intervals based on fitting a Cox model to the studies separately for the RFS and OS endpoints, and these estimates were reported in Kirkwood et al. [8] and Kirkwood et al. [9]. Table 1 also shows maximum likelihood estimates of the cure rate for both the RFS and OS endpoints based on fitting the model in equation (1). The results in Table 1 are thus based on a frequentist (i.e., non-Bayesian) analysis of E1684 and E1690. The results in Table 2 are for both RFS and OS based on *a*_0_ = 0, 0.4, 1 using the model in equation (A.1) in Additional file 1: Appendix A along with the prior in equation (2). We see from Table 2 how the estimates of the posterior hazard ratio and posterior cure rates change as more weight is given to the historical data for both the RFS and OS endpoints. Table 2 shows that when more weight is given to the historical data, the posterior hazard ratios increase, resulting in a greater percent reduction in relapse for the IFN arm compared to the observation arm. In addition, although not shown here for brevity, the HPD intervals for the hazard ratio also become narrower and do not include 1. This is reasonable since the posterior hazard ratios based on E1684 alone were much larger than E1690 alone, and therefore as more weight is given to E1684, the greater the posterior hazard ratios and the narrower the HPD intervals. Similarly, the posterior estimates of the cure rates in Table 2 become smaller as *a*_0_ is increased. This again can be explained by the fact that the posterior cure rates for each arm in E1684 alone were much smaller than those based on E1690 alone. The value *a*_0_ = 1 implies that the E1684 and E1690 data are equally weighted in the Bayesian analysis, and this value achieves the greatest percent reduction in relapse (34%) and overall survival (14%), and the smallest cure rates for both treatment arms for both RFS and OS.

**Table 2:** Bayesian analysis of E1690 using E1684 as historical data

**Endpoint**	**Weight (*a***_**0**_**)**	**Posterior hazard ratio**	**95% HPD Interval**	**% Reduction**	**Posterior cure rate estimate (95% HPD Interval)**	
					**OBS**	**IFN**
RFS	0	1.29	(0.98, 1.63)	29	0.32	0.41
					(0.24, 0.39)	(0.32, 0.49)
	0.4	1.32	(1.04, 1.62)	32	0.30	0.40
					(0.23, 0.36)	(0.33, 0.46)
	1	1.34	(1.10, 1.60)	34	0.28	0.39
					(0.23, 0.33)	(0.33, 0.44)
OS	0	1.01	(0.74, 1.30)	1	0.50	0.50
					(0.42, 0.58)	(0.42, 0.58)
	0.4	1.08	(0.82, 1.34)	8	0.44	0.46
					(0.36, 0.51)	(0.38, 0.54)
	1	1.14	(0.91, 1.38)	14	0.39	0.43
					(0.32, 0.46)	(0.36, 0.50)

**Figure 3:**
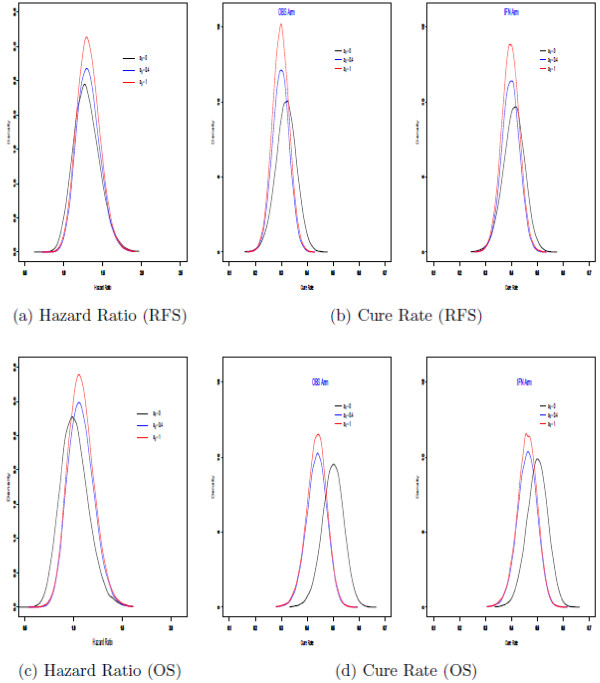
Plots of posterior densities of hazard ratios (a and c) and cure rates (b and d) for the full Bayesian analysis.

Figure 2 shows the posterior distributions of the hazard ratios and cure rates for separate Bayesian analyses of E1684 and E1690. Plots (a) and (c) in Figure 2 show the posterior distributions of the hazard ratios based on separate Bayesian analyses for E1684 and E1690 for the RFS and OS endpoints, respectively, and Plots (b) and (d) in Figure 2 show posterior estimates of the cure rates for RFS and OS, by treatment arm, based on separate analyses of E1684 and E1690. Plots (a) – (d) in Figure 2 essentially mimic the results based on the separate analyses conducted in [8,9]. Plots (a) – (d) in Figure 3 are based on the Bayesian analysis which combines the two studies via the power prior in equation (2). Plots (a) and (c) in Figure 3 show the posterior densities of the hazard ratio for three values of *a*_0_ for the RFS (Figure 3a) and OS (Figure 3c) endpoints. From these plots, we can see how the posterior distributions shift to the right and become more peaked as more weight (i.e., increasing *a*_0_) is given to the historical data. Specifically, as *a*_0_ is increased, we see a sharper peak in the posterior distribution of the hazard ratio in Figure 3a and c, thus giving a greater strength of evidence that the hazard ratio is larger than 1 (1.34 for RFS, 1.14 for OS). The same phenomenon occurs n Figure 3b and d which shows the posterior distributions of the cure rates for RFS and OS. In Figure 3b, we see that as *a*_0_ is increased, there is a shift to the left and sharper peaks in the posterior distributions of the cure rates, yielding a greater strength of evidence that the cure rates are 0.28 and 0.39 for the observation and IFN arms for RFS, respectively (Figure 3b, and 0.39 and 0.43 for the observation and IFN arms for OS, respectively (Figure 3d).

**Figure 4:**
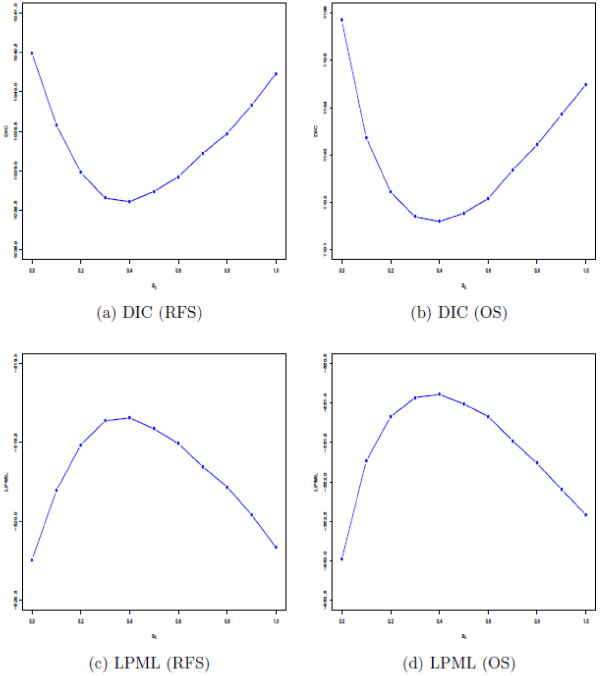
DIC and LPML plots for E1684 and E1690.

A critical issue is the choice of *a*_0_ in the power prior, that is, what value of *a*_0_ should the investigator use in the analysis? To address this issue, we use the value of *a*_0_ that yields the best model fit according to some Bayesian criterion, such as the Deviance Information Criterion (DIC) (see Additional file 1: Appendix B). Figure 4a and b shows that the DIC is optimized when *a*_0_ = 0.4 for both the RFS and OS endpoints suggesting that the Bayesian model fits best when *a*_0_ = 0.4 is used for both of these endpoints. Interestingly enough, the same optimal value of *a*_0_ = 0.4 is obtained under a different Bayesian criterion (Logarithm of the Pseudo Marginal Likelihood (LPML) (see Additional file 1: Appendix B and Figure 4c and d). Thus, the value of *a*_0_ = 0.4 appears to be a very reasonable value to use for the Bayesian analysis, along with other *a*_0_ values used as a sensitivity analysis, such as *a*_0_ = 0 and *a*_0_ = 1.

We compared our Bayesian analysis based on the power prior to three other standard frequentist methods as shown in Table 3. These are i) a Cox regression analysis on the pooled E1684 and E1690 data along with a binary study covariate in the model, ii) a Cox regression analysis of the pooled data without a study covariate, and iii) a gamma frailty model treating study as a cluster (2 clusters). Methods i), ii) and iii) yielded similar results as the Bayesian analysis corresponding to *a*_0_ = 1 in Table 2 for both RFS and OS showing that our Bayesian analysis using *a*_0_ = 1 essentially yields the same results as these three frequentist methods as well as being a more flexible methodology for incorporating historical data. However, we note that *a*_0_ = 1 is not an optimal value for the Bayesian analysis as shown by Figure 4. Our Bayesian methodology proposed here is much more general and more flexible than a frequentist analysis and chooses an optimal value of *a*_0_ based on the best model fit to the data.

**Table 3:** Maximum likelihood analysis of E1690 and E1684

**Endpoint**	**Weight (*a***_**0**_**)**	**Hazard ratio estimate**	**95% CI**	**% Reduction**	**P-value**
RFS	Study covariate	1.35	(1.12, 1.63)	35	0.0018
	Combined	1.34	(1.11, 1.62)	34	0.0021
	Frailty	1.35	(1.12, 1.62)	35	0.0019
OS	Study covariate	1.15	(0.93, 1.41)	14	0.196
	Combined	1.13	(0.92, 1.39)	13	0.232
	Frailty	1.14	(0.93, 1.40)	14	0.200

## Discussion

The upward shifts in the OBS and IFN arm in E1690 relative to E1684 do not have a clear cut explanation. It was conjectured that the standard of care improved with time, therefore resulting in improved RFS and OS in E1690. We carried out several analyses to assess this conjecture by fitting a time trend in the Cox model. As noted in Section 5, this time trend effect was highly non-significant in all models fit. Another analysis examining institutional effects was also conducted and the analysis yielded non-significant institutional effects. Another issue still very difficult to explain is why OS was much better for the OBS patients in E1690 than in E1684.

As noted earlier, the KM plot for OS showed almost total overlap between the IFN and OBS arms. One potential explanation for this is that patients on the OBS arm received salvage therapies after relapse that may have improved their OS. The inclusion of these salvage therapies was not accounted for in the OS analysis. It will take further confirmatory trials to get better answers to these two unresolved issues.

**Figure 5:**
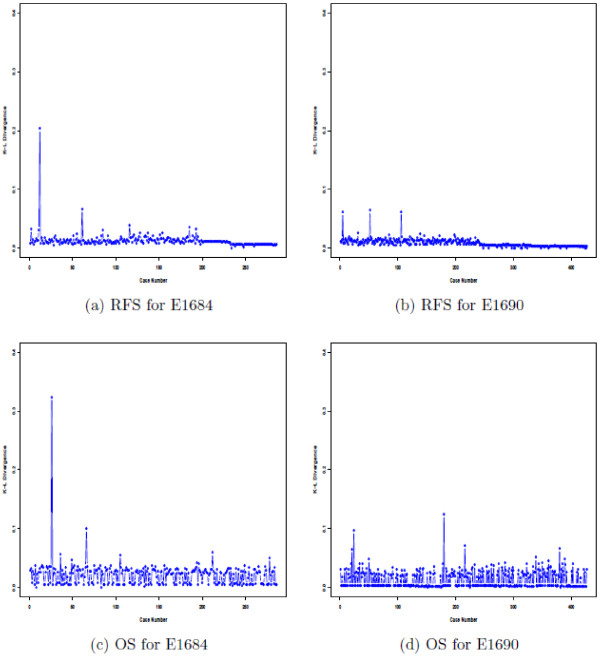
**RFS and OS plots of the KL divergence between the posterior for the full data and the posterior without the *i***^***th ***^**case for E1684 and E1690.**

**Figure 6:**
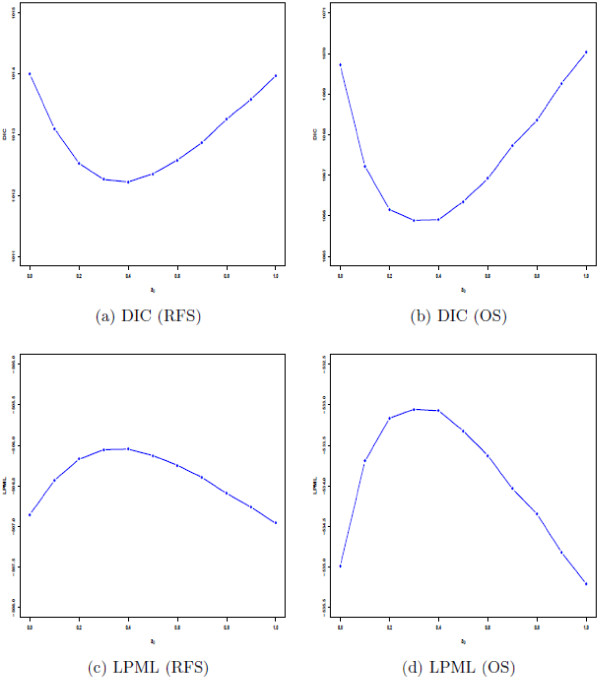
DIC and LPML plots for E1684 and E1690 after excluding the influential cases.

All of the computations for the models that were presented here were done using a FORTRAN 95 program, but the same results can also be easily obtained and programmed using PROC MCMC in SAS. An important question here is that of the robustness of the choice of *a*_0_ in the presence of outliers or influential points in the data. To address this issue, we carried out a case influence analysis using the methods of Cho et al. [12]. Figure 5 shows the RFS and OS plots of the KL divergence between the posterior for the full data and the posterior without the *i*^*th*^ case for E1684 and E1690. From Figure 5, we see that the values of the KL divergence were small for all observations. For the E1864 data, the maximum values of the KL divergence were 0.203 for RFS and 0.324 for OS, respectively, and there was only one additional case in which the KL divergence value was greater than 0.06 for each of RFS and OS. For the E1690 data, there were three cases for RFS and five cases for OS, in which the KL divergence values were greater than 0.06. We deleted these relatively most influential cases (two for E1684 and three for E1690 for RFS and two for E1684 and five for E1690 for OS) and then re-ran the analysis to obtain the optimal values of *a*_0_ under DIC and LPML for RFS and OS. Figure 6 shows the DIC and LPML plots for E1684 and E1690 after excluding these influential cases. We see from this figure that the optimal choice of *a*_0_ remains to be 0.4 for both RFS and OS. Thus, the optimal choice of *a*_0_ under DIC and LPML is relatively robust to influential observations.

Moreover, we have carried out a sensitivity analysis with respect to the choice of the initial prior, using several possible choices of initial priors. To this end, we consider *π*_0_(*β*, *λ*) = *π*_0_(*β*)*π*_0_(*λ*) where *β* ~ *N*(0, *τ*_0_^2^*I*_2_), *I*_2_ is the 2 × 2 identity matrix, and λ_j_ ~ Gamma (*b*_01_, *b*_02_) (j = 1, 2, …, J), where J=5 for RFS and J=10 for OS, with density proportional to $\lambda_{j}^{\left(b_{01}-1\right)}\text{exp}\left(-b_{02}\lambda_{j}\right)$ and *b*_01_ ≥ 0 and *b*_02_ ≥ 0. For RFS, the posterior hazard ratios and 95% HPD intervals for the treatment effect were 1.294 and (0.977, 1.626) for *a*_0_ = 0, 1.320 and (1.033, 1.611) for *a*_0_ = 0.4, and 1.346 and (1.109, 1.616) for *a*_0_ = 1 when *τ*_0_^2^ = 10 and *b*_01_ = *b*_02_ = 1; and 1.293 and (0.968, 1.628) for *a*_0_ = 0, 1.320 and (1.037, 1.616) for *a*_0_ = 0.4, and 1.342 and (1.098, 1.597) for *a*_0_ = 1 when *τ*_0_^2^ = 2 and *b*_01_ = *b*_02_ = 10. For OS, the posterior hazard ratios and 95% HPD intervals for the treatment effect were 1.012 and (0.726, 1.303) for *a*_0_ = 0, 1.081 and (0.832, 1.352) for *a*_0_ = 0:4, and 1.138 and (0.910, 1.371) for *a*_0_ = 1 when *τ*_0_^2^ = 10 and *b*_01_ = *b*_02_ = 1; and 1.017 and (0.742, 1.317) for *a*_0_ = 0, 1.081 and (0.826, 1.350) for *a*_0_ = 0.4, and 1.135 and (0.909, 1.371) for *a*_0_ = 1 when *τ*_0_^2^ = 2 and *b*_01_ = *b*_02_ = 10. These results were quite similar to those given in Table 2. Other choices of (*τ*_0_^2^, *b*_01_, *b*_02_) were also tried and the results were similar. Thus, the analysis of E1684 and E1690 is quite robust to the choice of the initial prior.

## Conclusions

We have presented a Bayesian analysis of E1684 and E1690 using the ideas of the power prior. This prior incorporates historical data in a natural way and gives the investigator a great deal of control over the weight given to the historical data through the parameter *a*_0_. In Section 5, we carried out a detailed Bayesian analysis using this prior and observed that the analysis provided a more coherent, flexible, and potentially more accurate analysis than a separate analysis of these data or a frequentist analysis of these data. The methodology provides a consistent framework for carrying out a single unified analysis by combining data from two studies. Our analysis showed that using *a*_0_ = 0.4 yielded the best fitting model based on DIC and LPML, therefore suggesting that this value is the optimal value in discounting the E1684 data in the Bayesian analysis of E1690. The Bayesian analysis using *a*_0_ = 0.4 yielded markedly different results than those of *a*_0_ = 0 and *a*_0_ = 1 in terms of estimated hazard ratios and reductions in relapses and/or deaths in using IFN as compared to OBS. A philosophical issue that arises here is that when such an analysis is to be carried out. The Bayesian analysis of E1690 was conducted after seeing the inconsistent results between the studies. A more appropriate approach would be to specify these Bayesian analyses in the protocol itself before the E1690 data is even collected. Such a decision would also impact the design of E1690.

## Competing interests

The authors declare that they have no competing interests.

## Authors’ contributions

JGI proposed the conception and design, provided financial and administrative support, and collected the data. MHC carried out the data analysis. All authors participated in the interpretation and manuscript writing, and revision. All authors read and approved the final manuscript.

## Pre-publication history

The pre-publication history for this paper can be accessed here:

http://www.biomedcentral.com/1471-2288/12/183/prepub

## Supplementary Material

Additional file 1: Appendix A The Likelihood Function, Prior, and Posterior, **Appendix B:** Bayesian Model Comparison Criteria. Click here for file
