# Supplementary material for: Bayesian methods in clinical trials: a Bayesian analysis of ECOG trials E1684 and E1690
Source: BMC Med Res Methodol. 2012 Nov 29;12:183. doi: 10.1186/1471-2288-12-183 (PMC3571975; doi:10.1186/1471-2288-12-183)
Supplement: Additional file 1: Appendix A — The Likelihood Function, Prior, and Posterior, Appendix B: Bayesian Model Comparison Criteria. [file 1471-2288-12-183-S1.pdf]

Supplementary Materials for
"Bayesian Methods in Clinical Trials: A Bayesian Analysis of ECOG Trials E1684 and E1690"

Joseph G. Ibrahim, Ming-Hui Chen, and Haitao Chu

Appendix A: The Likelihood Function, Prior, and Posterior

Suppose we have n patients in the current trial (E1690). Let y_i denote the survival time for the ith patient, which may be right censored, and let ν_i denote the censoring indicator, which equals 1 if y_i is a failure time and 0 if it is right censored. Also let $\boldsymbol{x}_i = (1, \text{trt}_i)'$ denote the vector of covariates, where trt_i denotes the treatment indictor such that $\text{trt}_i = 1$ if the ith patient received IFN and $\text{trt}_i = 0$ if the ith patient received OBS. The observed current data is $D = (n, \boldsymbol{y}, \boldsymbol{\nu}, X)$, where $\boldsymbol{y} = (y_1, \ldots, y_n)'$, $\boldsymbol{\nu} = (\nu_1, \ldots, \nu_n)'$, $X = (\boldsymbol{x}_1, \ldots, \boldsymbol{x}_n)'$. Following Chen et al. (1999), we obtain the likelihood function as follows:

$$L(\boldsymbol{\beta}, \boldsymbol{\lambda}|D) = \prod_{i=1}^{n} \Big\{ \exp(\boldsymbol{x}_i'\boldsymbol{\beta}) f_0(y_i|\boldsymbol{\lambda}) \Big\}^{\nu_i} \exp\{-\exp(\boldsymbol{x}_i'\boldsymbol{\beta}) F_0(y_i|\boldsymbol{\lambda}))\}, \tag{A.1}$$

where $\boldsymbol{\beta} = (\beta_0, \beta_1)'$, and $F_0(y|\boldsymbol{\lambda})$ is the cumulative distribution function and $f_0(y|\boldsymbol{\lambda})$ is the corresponding density function. In (A.1), we assume a piecewise exponential model for $F_0(y|\boldsymbol{\lambda})$, which is given by

$$F_0(y|\boldsymbol{\lambda}) = 1 - \exp\Big\{ -\lambda_j(y - s_{j-1}) - \sum_{g=1}^{j-1} \lambda_g (s_g - s_{g-1}) \Big\},$$

where $s_{j-1} \leq y < s_j$, $s_0 = 0 < s_1 < s_2 < \ldots < s_J = \infty$, and $\boldsymbol{\lambda} = (\lambda_1, \ldots, \lambda_J)'$. We note that in (A.1), the cure rates are $\exp\{-\exp(\beta_0 + \beta_1)\}$ and $\exp\{-\exp(\beta_0)\}$ for patients in the IFN arm and the OBS arm, respectively.

Suppose we have n_0 patients in the historical trial (E1684). Let y_{0i} denote the survival time for the ith patient, which may be right censored, and let ν_{0i} denote the censoring

indicator, which equals 1 if y_{0i} is a failure time and 0 if it is right censored. Also let $\boldsymbol{x}_{0i} = (1, \mathrm{trt}_{0i})'$ denote the vector of covariates, where trt_{0i} denotes the treatment indictor for the ith patient in the historical trial. The observed historical data is $D_0 = (n_0, \boldsymbol{y}_0, \boldsymbol{\nu}_0, X_0)$, where $\boldsymbol{y}_0 = (y_{01}, \ldots, y_{0n})'$, $\boldsymbol{\nu}_0 = (\nu_{01}, \ldots, \nu_{0n})'$, $X_0 = (\boldsymbol{x}_{01}, \ldots, \boldsymbol{x}_{0n})'$. Then, the power prior in Equation (2) based on the historical data D_0 is given by

$$\pi(\boldsymbol{\beta}, \boldsymbol{\lambda}|D_0, a_0) \propto \Big[\prod_{i=1}^{n_0} \Big\{ \exp(\boldsymbol{x}_{0i}'\boldsymbol{\beta}) f_0(y_{0i}|\boldsymbol{\lambda}) \Big\}^{\nu_{0i}} \exp\{-\exp(\boldsymbol{x}_{0i}'\boldsymbol{\beta}) F_0(y_{0i}|\boldsymbol{\lambda}))\}\Big]^{a_0} \pi_0(\boldsymbol{\beta}, \boldsymbol{\lambda}), \tag{A.2}$$

where $0 \leq a_0 \leq 1$ and $\pi_0(\boldsymbol{\beta}, \boldsymbol{\lambda})$ is an initial prior. We specify a noninformative uniform initial prior for $\boldsymbol{\beta}$ and $\boldsymbol{\lambda}$. Specifically, $\pi_0(\boldsymbol{\beta}, \boldsymbol{\lambda}) \propto 1$.

Let $\boldsymbol{\theta} = (\boldsymbol{\beta}, \boldsymbol{\lambda})$. Using (A.1) and (A.2), the posterior distribution of $\boldsymbol{\theta}$ given (D, D_0, a_0) can be written as follows:

$$\pi(\boldsymbol{\theta}|D, D_0, a_0) \propto L(\boldsymbol{\beta}, \boldsymbol{\lambda}|D)\pi(\boldsymbol{\beta}, \boldsymbol{\lambda}|D_0, a_0), \tag{A.3}$$

where $\pi(\boldsymbol{\beta}, \boldsymbol{\lambda}|D_0, a_0)$ is defined by (A.3). Chen et al. (1999) developed an efficient Markov chain Monte Carlo sampling algorithm to sample from $\boldsymbol{\theta}$ from the posterior distribution (A.3).

Appendix B: Bayesian Model Comparison Criteria

To determine an optimal combination of (J, a_0) in the posterior distribution (A.3), we consider two Bayesian model comparison criteria, namely, the deviance information criterion (DIC) and the Logarithm of Pseudo Marginal Likelihood (LPML).

For DIC, we first define the deviance function as

$$\mathrm{Dev}(\boldsymbol{\theta}) = -2 \log L(\boldsymbol{\beta}, \boldsymbol{\lambda}|D),$$

where $L(\boldsymbol{\beta}, \boldsymbol{\lambda}|D)$ is defined in (A.1). Let $\bar{\boldsymbol{\theta}} = E[\boldsymbol{\theta}|D, D_0, a_0]$ and $\overline{\mathrm{Dev}} = E[\mathrm{Dev}(\boldsymbol{\theta})|D, D_0, a_0]$ denote the posterior means of $\boldsymbol{\theta}$ and $\mathrm{Dev}(\boldsymbol{\theta})$ with respect to the posterior distribution in

(A.3), respectively. Then, according to Spiegelhalter et al. (2002), the DIC measure is defined as

$$\text{DIC} = \text{Dev}(\bar{\boldsymbol{\theta}}) + 2p_D, \tag{A.4}$$

where $p_D = \overline{\text{Dev}} - \text{Dev}(\bar{\boldsymbol{\theta}})$ is the effective number of model parameters. The first term in (A.4) measures the goodness-of-fit. The smaller the $\text{Dev}(\bar{\boldsymbol{\theta}})$, the better the fit. The second term $2p_D$ in (A.4) is the dimension penalty. The DIC in (A.4) is a Bayesian measure of predictive model performance, which is decomposed into a measure of fit ($\text{Dev}(\bar{\boldsymbol{\theta}})$) and a measure of model complexity (p_D). The smaller the value the better the model will predict new observations generated in the same way as the data. The form of DIC given in (A.4) is exactly the same as AIC. However, unlike AIC, the effective number of model parameters (p_D) is automatically calculated according to the posterior distribution (A.3).

The conditional predictive ordinate (CPO) in model comparison is a Bayesian cross-validation approach. Given the model defined by (A.1), the CPO statistic for the ith patient is defined as

$$\text{CPO}_i = E\Big[\Big\{\exp(\boldsymbol{x}_i'\boldsymbol{\beta}) f_0(y_i|\boldsymbol{\lambda})\Big\}^{\nu_i} \exp\{-\exp(\boldsymbol{x}_i'\boldsymbol{\beta}) F_0(y_i|\boldsymbol{\lambda}))\}|D_{(-i)}, D_0, a_0\Big],$$

where $D_{(-i)}$ is the observed data D with the ith patient removed and the expectation is taken with respect to the posterior distribution in (A.3) with D replaced by $D_{(-i)}$. This statistic is the posterior predictive probability of the ith observation given all other observed data under the assumed model. The larger the value of CPO_i, the more the ith observation supports the fitted model. According to Chen et al. (2000), CPO_i can be computed as

$$\text{CPO}_i = \Big\{E\Big(\Big[\Big\{\exp(\boldsymbol{x}_i'\boldsymbol{\beta}) f_0(y_i|\boldsymbol{\lambda})\Big\}^{\nu_i} \exp\{-\exp(\boldsymbol{x}_i'\boldsymbol{\beta}) F_0(y_i|\boldsymbol{\lambda}))\}\Big]^{-1}\Big|D, D_0, a_0\Big)\Big\}^{-1},$$

where the expectation is taken with respect to the joint posterior distribution of $\boldsymbol{\theta}$ given by (A.3). The CPO_i value can be summed over all patients to form a single summary statistics — logarithm of pseudo marginal likelihood (LPML) (Ibrahim et al., 2001) given

by

$$\text{LPML} = \sum_{i=1}^{n} \log \text{CPO}_i.$$

Models with larger LPML values are preferred to models with lower LPML values. According to Gelfand and Dey (1994), LPML implicitly includes a similar dimensional penalty as AIC asymptotically.

We use the DIC and LPML criteria to determine the optimal choice of (J, a_0). For E1684 and E1690, the optimal choices of (J, a_0) were $(J = 5, a_0 = 0.4)$ for RFS and $(J = 10, a_0 = 0.4)$ for OS according to both DIC and LPML.

Additional References

Chen, M.-H., Ibrahim, J. G., and Sinha, D. (1999). A New Bayesian Model For Survival Data With a Surviving Fraction. *Journal of the American Statistical Association* **94**, 909-919.

Chen, M.-H., Shao, Q.-M., and Ibrahim, J. G. (2000). *Monte Carlo Methods in Bayesian Computation*. New York: Springer.

Gelfand, A. E. and Dey, D. K. (1994). Bayesian model choice: Asymptotics and exact calculations. *Journal of the Royal Statistical Society, Series B* **56**, 501-514.

Spiegelhalter, D. J., Best, N. G., Carlin, B. P., and van der Linde, A. (2002). Bayesian measures of model complexity and fit (with Discussion). *Journal of the Royal Statistical Society, Series B* **64**, 583-639.
